# β-tricalcium phosphate/calcium sulfate loaded with contezolid acefosamil (MRX-4) for antimicrobial potency, prevention and killing efficacy of MRSA biofilm

**DOI:** 10.3389/fphar.2025.1657099

**Published:** 2025-09-23

**Authors:** Nan Jiang, Xin Zhang, Zi-Xian Liu, Hao-Yang Wan, Mou-Zhang Huang, Qing-Rong Lin, Guan-Qiao Liu, Peng Chen, Bin Yu

**Affiliations:** ^1^ Guangdong Institute of Orthopaedics & Traumatology, Nanfang Hospital, Southern Medical University, Guangzhou, China; ^2^ Guangdong Key Laboratory of Bone and Cartilage Regenerative Medicine, Nanfang Hospital, Southern Medical University, Guangzhou, China; ^3^ Department of Trauma Emergency Center, Ganzhou Hospital-Nanfang Hospital, Southern Medical University, Ganzhou, China; ^4^ Department of Orthopaedics and Traumatology, Wuyi Hospital of Traditional Chinese Medicine, Jiangmen, China; ^5^ Department of Orthopaedics, Lanzhou University Second Hospital, Lanzhou, China; ^6^ Department of Orthopaedics, Hainan General Hospital, Hainan Hospital affiliated to Hainan Medical University, Haikou, China

**Keywords:** calcium sulfate, β-tricalcium phosphate/calcium sulfate, MRX-4, MRSA biofilm, in-vitro experiment

## Abstract

**Objectives:**

This study aimed to evaluate the antimicrobial potency and duration of contezolid acefosamil (MRX-4) combined with gentamicin against methicillin-resistant *Staphylococcus aureus* (MRSA) biofilms *in vitro*. We also compared its performance, when delivered via calcium sulfate (CS) and β-tricalcium phosphate/calcium sulfate (β-TCP/CS) carriers, with the conventional vancomycin + gentamicin regimen.

**Methods:**

Antibiotic-loaded beads containing MRX-4 + gentamicin (C + G) or vancomycin + gentamicin (V + G) were prepared using CS and β-TCP/CS carriers. Antimicrobial potency and release duration were assessed using a modified Kirby-Bauer zone of inhibition (ZOI) assay. MRSA biofilm prevention and eradication were evaluated through colony forming unit (CFU) counting and confocal laser scanning microscopy (CLSM).

**Results:**

C + G demonstrated prolonged antimicrobial activity, maintaining effective ZOIs for at least 40 days, whereas V + G lost activity by day 40 (P < 0.05). Both C + G and V + G significantly prevented biofilm formation and reduced CFUs by > 8 logs (P < 0.001), with no significant difference between carrier types. In biofilm eradication assays, both treatments reduced CFUs by 3–4 logs; however, C + G showed superior efficacy over V + G at day 3 (P < 0.01). CLSM confirmed substantial biofilm disruption and bacterial killing in C + G-treated groups.

**Conclusion:**

MRX-4 combined with gentamicin, delivered via CS and β-TCP/CS carriers, exhibits superior and sustained local antimicrobial efficacy compared to vancomycin, particularly in eradicating MRSA biofilms.

## 1 Introduction

Bone infections are severe conditions that occur when bacteria or microbes infiltrate bone tissue, leading to high rates of illness, extended treatment periods, and a considerable risk of recurrence (Thabit et al., 2019). Osteomyelitis often originates from injuries, post-operative issues, bloodborne spread, or conditions like diabetes (Wang X. et al., 2023). Symptoms include localized pain, swelling, and the development of fistulas, with severe cases potentially causing progressive bone damage, deformities, and loss of function (Bury et al., 2021). Diabetic foot osteomyelitis (DFO), another widespread bone infection, is linked to diabetic peripheral neuropathy, microvascular issues, and a weakened immune system (Armstrong et al., 2023; Senneville et al., 2024). These physiological disruptions contribute to poor wound healing, excessive bacterial growth, and deep bone infection, making DFO a primary contributor to non-traumatic lower limb amputations and greatly impacting the quality of life for patients (Tardáguila-García et al., 2021).

Currently, osteomyelitis is managed primarily through a combination of systemic antibiotic therapy and surgical interventions, such as debridement, sequestrectomy, local drainage, and defected tissue reconstruction (Wang X. et al., 2023). Nonetheless, the impaired blood supply to infected bone tissue hinders the efficacy of systemically administered antibiotics, thereby rendering complete bacterial elimination extremely difficult (Li K. et al., 2023). This challenge is further exacerbated in cases of chronic osteomyelitis and diabetic foot infections, where biofilm formation by multidrug-resistant (MDR) bacteria significantly compromises treatment efficacy (Wang et al., 2024; Yang et al., 2024). In this context, local antibiotic delivery systems (LADS) have emerged as an innovative solution, aiming to achieve high local antibiotic concentrations at the infection site, improve bacterial clearance, and reduce systemic toxicity (W et al., 2021).

Calcium sulfate (CS), among available LADS, has garnered widespread attention for its biocompatibility, biodegradability, and osteoconductive properties (Shi et al., 2022). CS granules or implants can gradually release antibiotics at the infection site while degrading into calcium ions and water, offering a temporary scaffold for bone regeneration (Li X.-D. et al., 2023). In comparison to traditional polymethylmethacrylate (PMMA)-based cements, CS fully degrades, negating the need for surgical removal and thereby decreasing patient discomfort (Steadman et al., 2023). Additionally, CS is capable of effectively delivering a broad spectrum of antibiotics, including heat-sensitive compounds, at higher local concentrations, positioning it as a promising option for treating osteomyelitis and DFO (Xu et al., 2022; Peterson et al., 2021).

Despite its benefits, CS also faces several challenges. Initially, its relatively swift degradation can exceed the pace of bone regeneration, potentially leaving behind infection risks post-CS resorption. Secondly, while CS facilitates the release of high-concentration antibiotics, Maintaining a stable and extended antibacterial effect is particularly challenging in chronic osteomyelitis and MRSA-related infections, where biofilm development can shield bacteria from the effects of antibiotics (Masters et al., 2022). Research has shown that antibiotic-laden scaffolds, while widely used in clinical settings, have limited efficacy against biofilm infections caused by gram-positive bacteria such as MRSA (Chen et al., 2023). Furthermore, the emergence of MDR bacteria, including MRSA and vancomycin-resistant *Enterococcus* (VRE), notably heightens the complexity of effective treatment (Jernigan et al., 2020). These issues underscore the necessity for innovative antibiotics and combined therapies that boost antimicrobial effectiveness, ensure sustained drug delivery, and enhance osteomyelitis treatment outcomes.

MRSA, a primary pathogen of chronic osteomyelitis and DFO, possesses the capacity to produce biofilms, persists within infected bone tissue, and circumvent traditional antibiotic therapy (Guo et al., 2024). Currently, vancomycin serves as the first-line antibiotic for treating MRSA-related infections; however, it has significant drawbacks, such as nephrotoxicity, inadequate bone penetration, and a brief duration of release when employed in local antibiotic delivery systems (Steinmetz et al., 2015). Additionally, the incidence of vancomycin resistance is on the rise, with the emergence of vancomycin-intermediate *S. aureus* (VISA) and vancomycin-resistant *S. aureus* (VRSA), further complicating treatment strategies (Russo and Venditti, 2021; Rao et al., 2022). Given the pressing challenges of antibiotic resistance and the need for effective treatments, there is an urgent necessity to develop innovative antibiotics that exhibit improved antimicrobial potency, superior bone tissue penetration, and a significantly reduced propensity for resistance emergence.

Oxazolidinones have recently gained recognition as a promising class of antibiotics for treating MRSA infections, owing to their unique mechanism of action (Roger et al., 2018). Linezolid, the first FDA-approved oxazolidinone, has demonstrated broad-spectrum activity against multidrug-resistant Gram-positive bacteria, including resistant strains such as methicillin-resistant *Staphylococcus aureus* (MRSA), vancomycin-resistant enterococci (VRE), and others, as evidenced by its clinical trial performance and effectiveness in treating infections such as community-acquired pneumonia in children (Kato et al., 2021; Korang et al., 2021). However, its long-term use is significantly limited by severe adverse effects, including bone marrow suppression, peripheral neuropathy, and lactic acidosis, restricting its suitability for prolonged treatment of bone infections (Veerman et al., 2023; Xerri et al., 2015). This has driven the development of next-generation oxazolidinones with improved safety and efficacy.

MRX-4 (contezolid acefosamil) is a novel second-generation oxazolidinone. MRX-4 is a prodrug of contezolid, which can be converted to MRX-1352 both *in vivo* and *in vitro*, and subsequently transformed into contezolid. MRX-4 and MRX-1352 has no antibacterial activity by themself. Their active metabolite, contezolid (MRX-1), which demonstrates excellent antibacterial activity, has been approved by the China National Medical Products Administration (NMPA) for the treatment of complicated skin and soft tissue infections (cSSTI) (Hoy, 2021). Studies have demonstrated MRX-1’s potent anti-MRSA activity, positioning it as a promising candidate for bone infection treatment (Zhang et al., 2024). Compared to linezolid, MRX-1 exhibits enhanced safety, greater tolerability, and reduced hematologic toxicity, allowing for long-term administration with fewer adverse effects (Zhao et al., 2022; Almeida et al., 2023). Furthermore, MRX-1 has a broad antibacterial spectrum, covering MRSA, VRE, and other MDR pathogens, which further expands its clinical utility (Wang et al., 2021). Notably, MRX-4 has been designed as an O-acyl phosphoramidate prodrug to significantly improve aqueous solubility, exceeding 200 mg/mL—vastly higher than the approximately 0.2 mg/mL solubility of contezolid—thereby facilitating its diffusion through the extracellular polymeric substance (EPS) matrix of biofilms (Liu et al., 2022). This elevated solubility may enable MRX-4 to penetrate biofilm layers more effectively, and once inside the biofilm, MRX-4 can be converted to the active MRX-1, achieving localized antibacterial effects (Wang X.-K. et al., 2023). This “prodrug diffusion followed by bioactivation” mechanism provides a plausible basis for why MRX-4 could outperform standard oxazolidinones in biofilm contexts.

At present, MRX-4 is in phase III trials for diabetic foot infections, showing considerable systemic effectiveness against MRSA and other MDR pathogens (Bulitta et al., 2024). However, its local use in bone has not been systematically evaluated; data on local antimicrobial performance, release kinetics, and compatibility with resorbable carriers remain limited. To address this translational gap, we examined a resorbable local strategy pairing MRX-4 (via its active metabolite MRX-1) with gentamicin. Relative to prior vancomycin- or linezolid-loaded carriers, this C + G approach offers (i) intracellular ribosomal activity (MRX-1) that may engage metabolically active biofilm subpopulations; (ii) pharmacologic complementarity with gentamicin’s rapid outer-layer bactericidal effect, potentially facilitating deeper penetration; and (iii) delivery via fully resorbable calcium sulfate (CS) or β-TCP/CS carriers that enable sustained local release within post-debridement dead space (Gogia et al., 2009; Jiang et al., 2021; Anagnostakos et al., 2008).

We therefore hypothesized that C + G, delivered by CS or β-TCP/CS, would outperform vancomycin + gentamicin (V + G) in eradicating established MRSA biofilms under dynamic refresh conditions. Accordingly, we evaluated sustained antimicrobial activity, quantitative drug release, and biofilm prevention/eradication *in vitro*, comparing CS and β-TCP/CS carriers, to clarify the potential of MRX-4 for localized therapy in osteomyelitis and diabetic foot osteomyelitis.

## 2 Materials and methods

### 2.1 Bacterial strains and culture condition

MRSA, ATCC43300, a standard *S. aureus* strain derived from our laboratory, was selected for this study. The resuscitated bacteria were inoculated into a 50 mL centrifuge tube containing 10 mL of trypsin-soybean broth (TSB; Pythonbio, Guangdong, China) with a half-tightened lid, tilted at a 45° angle, and placed on a shaking table at a constant temperature of 37 °C and 200 rpm, and cultured for 16 h.

### 2.2 Preparation of antibiotic-loaded beads (ALBs)

ALBs were prepared using Genex and Stimulan Rapid Cure products (Biocomposites Ltd., Staffordshire, United Kingdom). Genex is a synthetic biphasic material composed of β-tricalcium phosphate (β-TCP) and CS in a 1:1 weight ratio, while Stimulan is a high-purity CS product. The mixing ratios for the C + G and V + G groups were 800 mg of MRX-4 powder (Shanghai MicuRx Pharmaceutical Co. Ltd., Shanghai, China) with 240 mg of gentamicin powder (Tianxin Ltd., Guangdong, China), and 1000 mg vancomycin powder (Eli Lily and Co., Indiana, United States) with 240 mg of gentamicin powder, respectively, combined with 10 cc of either Genex or Stimulan powder. CS or GENEX can be diluted with saline, and after mixing with sterile saline, it solidifies within 3 min. We mixed the powders thoroughly in a biosafety cabinet and added 4 mL of sterile saline. The smooth paste was then pressed into a flexible mold with 4.8 mm wide hemispherical depressions. After waiting for complete solidification, the beads were selected and stored in a 4 °C refrigerator. Each bead weighed approximately 0.108 g.

### 2.3 Antibiotic-elution potency and duration for inhibition of planktonic bacteria

The revised Kirby-Bauer test was used to assess the potency of ALBs over time. Overnight cultures of MRSA (ATCC43300) were diluted to 1% in TSB medium, and 100 μL of the diluted medium was spread on tryptic soy agar (TSA, Pythonbio, Guangdong, China) plates. Three ALBs with C + G or V + G were placed on the agar surface using aseptic forceps, with each bead spaced an equal distance apart. The plates were then placed in a bacteria incubator set at 37 °C with 5% CO_2_. The diameter of the inhibition zone (ZOI) was measured at 24 h. The bacterial culture-medium was replaced daily, but the beads remained unchanged. The diameter of each inhibition zone was measured, and the corresponding area was calculated using the formula A = πr^2^, where A is the inhibition zone area, r is the radius (half of the measured diameter), and π is approximated as 3.142. The assay was considered complete when either (i) the inhibition zone diameter decreased to less than 6 mm, indicating loss of detectable antibacterial activity, or (ii) the residual bead diameter was reduced to less than 4.8 mm due to degradation.

### 2.4 Prevention of MRSA biofilm

ALBs and unloaded beads (five beads per well) were placed in 12-well plates (Corning, NY, United States) and confocal dishes with a diameter of 35 mm (Corning, NY, United States). Each well was inoculated with 2 mL of TSB containing 106 colony forming units (CFU)/mL. The dishes were placed in a bacterial incubator for overnight culture at 37 °C, 5% CO_2_, and 50 rpm. The culture medium was changed daily. The number of biofilm bacteria was observed after 1 and 3 days.

### 2.5 Killing of MRSA biofilm

Each well was inoculated with 2 mL of TSB containing 106 CFU/mL, without ALBs. The dishes were then placed in a bacterial incubator for overnight culture at 37 °C, with 5% CO_2_ and shaking at 50 rpm. The culture medium was changed daily. After 3 days, ALBs and unloaded beads were added, and the dishes were returned to the bacterial incubator for another overnight culture at 37 °C, 5% CO_2_, and 50 rpm. The number of biofilm bacteria was observed at 1 and 3 days.

### 2.6 Viable colony forming unit (CFU)

Each well was rinsed twice with 2 mL phosphate bufered saline (PBS) (Gibco, MD, United States) to remove planktonic cells. The surface of well was scraped using a cell scraper (Corning, NY, United States) to collect the biofilm bacteria into 1 mL PBS. Samples were vortexed for 20 s to homogenize the biofilm bacteria, tenfold serial dilutions were performed in PBS and plated onto TSA agar plates using the drop plate method, in triplicates. Wells with bacteria-inoculated media without any beads (control group 1, C1) or unloaded beads (control group 2, C2) were used as negative control.

### 2.7 Confocal laser scanning microscopy (CLSM)

Each well was inoculated with 2 mL of TSB containing approximately 10^6^ CFU/mL, without ALBs. The dishes were then placed in a bacterial incubator for overnight culture at 37 °C with 5% CO_2_ and shaking at 50 rpm. The culture medium was changed daily. After 3 days, ALBs and unloaded control beads were added, and the dishes were returned to the incubator for another overnight culture under the same conditions. To support the CFU data from the prevention assay, confocal laser scanning microscopy (CLSM; Olympus FV10i, Waltham, MA) was performed on biofilms grown in MatTek plates. Biofilms were observed at days 1 and 3. Each plate was rinsed with PBS (as described for the viable cell count), then stained with 1 µL SYTO9 (Thermo Fisher Scientific, United Kingdom) per 1 mL PBS for 20 min at room temperature, according to the manufacturer’s instructions. After staining, plates were gently rinsed with PBS once more, and 1 mL of PBS was added to each well before imaging. SYTO9 stains both live and dead cells green, enabling microscopic quantification of biofilm surface area. For each group, three CLSM images were acquired, and ImageJ (NIH) software was used to measure and statistically compare the biofilm surface areas.

### 2.8 Statistical analysis

All data were presented as the mean ± standard error (Mean ± SE) and were subjected to statistical analysis using GraphPad Prism 8 software. For comparisons between two groups, an independent samples t-test was employed when the sample size was greater than or equal to three and the data followed a normal distribution. If the variances were equal, a one-way ANOVA was initially used for statistical analysis. If the ANOVA results were statistically significant, *post hoc* Bonferroni tests were performed for pairwise comparisons. A P-value less than 0.05 was considered statistically significant, and statistical graphs were generated using GraphPad Prism 8 software. All results in this experiment were obtained from at least three independent control replicate experiments.

## 3 Results

### 3.1 Antibiotic elution potency and duration for inhibition of planktonic bacteria

**FIGURE 1:**
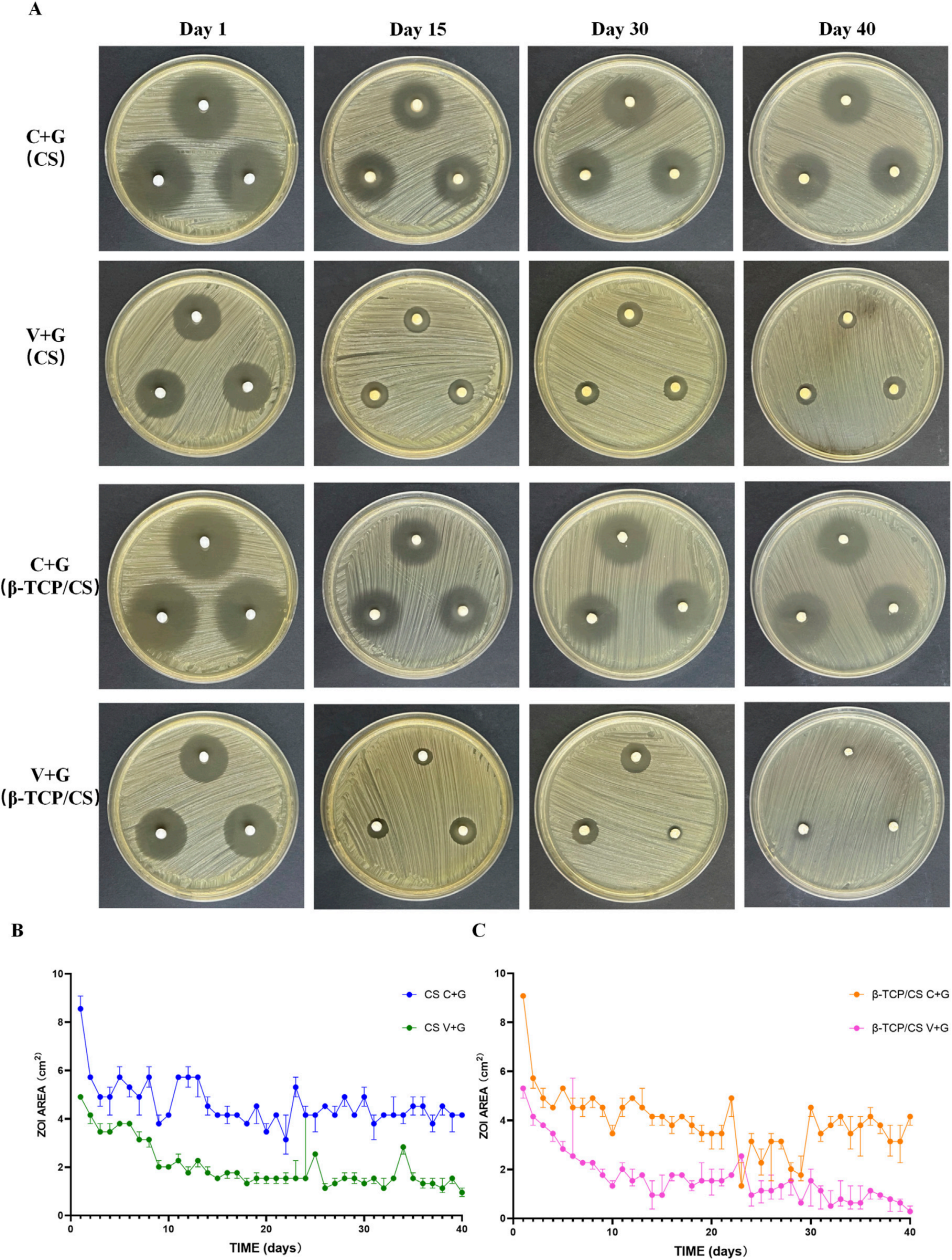
ZOIs of C + G versus V + G by CS and β-TCP/CS. Representative images of the ZOI assay showing the antimicrobial activity of MRX-4 + gentamicin (C + G) and vancomycin + gentamicin (V + G) released from CS and β-TCP/CS beads over time **(A)**. Statistical diagram comparing ZOI sizes between C + G and V + G over time **(B,C)**.

The antimicrobial potency and elution characteristics of C + G and V + G from CS and β-TCP/CS beads were evaluated by measuring the ZOI over time. The ATCC43310 strain was susceptible to both antibiotic combinations. However, the ZOI of V + G gradually decreased, reaching near zero by day 40, indicating a progressive loss of antimicrobial activity. In contrast, the ZOI of C + G remained stable after 14 days, fluctuating around the same level throughout the observation period. Even at the final stage of bead degradation, a substantial concentration of C + G was still being released, suggesting a prolonged antimicrobial effect (Figures 1A–C). Furthermore, no significant differences in ZOI were observed between CS and β-TCP/CS beads within the same antibiotic combination. This suggests that both carrier materials provided a comparable sustained release profile for the antibiotics, maintaining their antimicrobial efficacy over time (Supplementary Figure S1A,B).

### 3.2 Prevention of biofilm formation and eradication of established biofilms

To assess biofilm prevention and eradication efficacy, the TSA agar drop method was used for CFU counting in both control and experimental groups.

**FIGURE 2:**
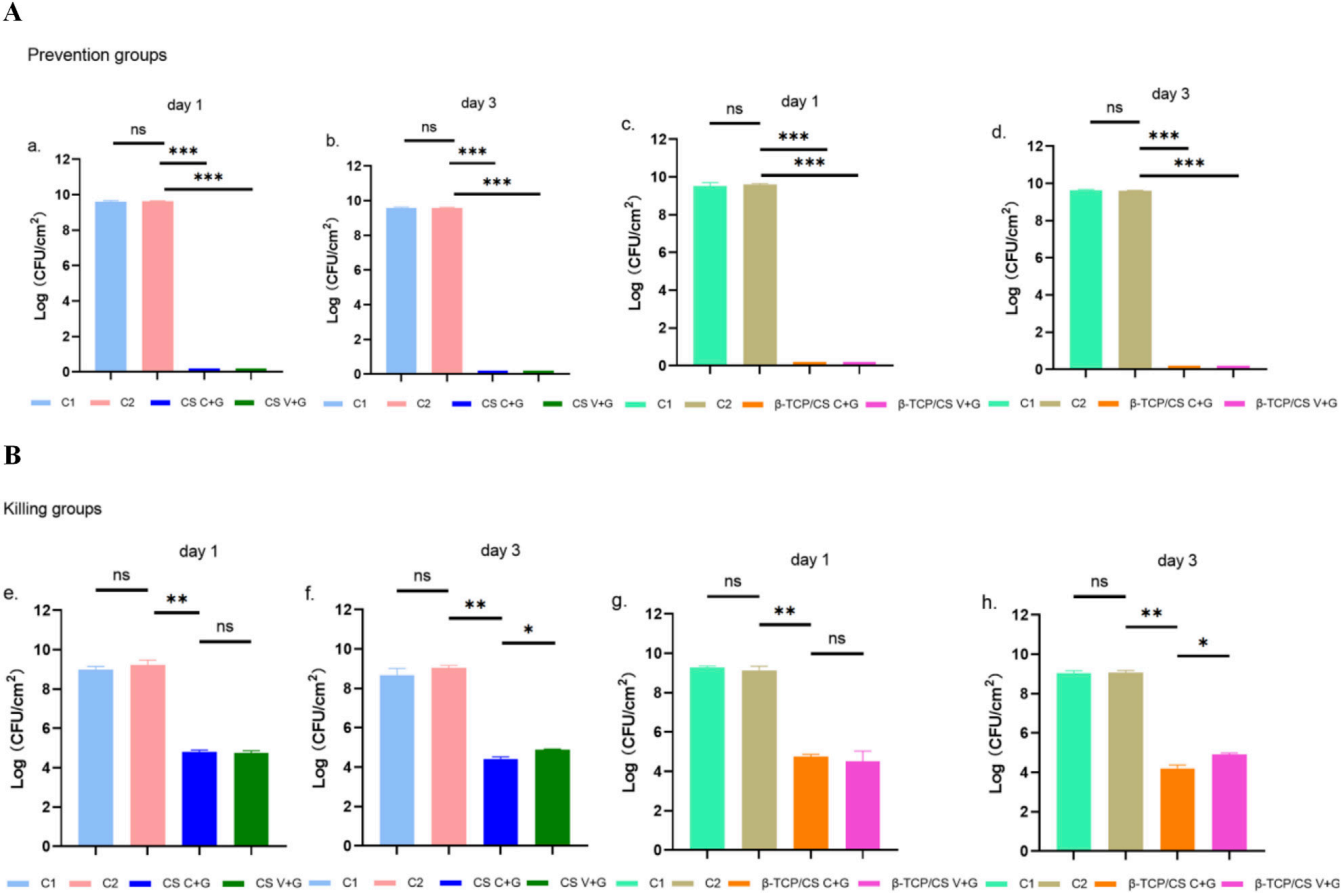
CS or β-TCP/CS Mixed with Antibiotics for the Prevention/Killing of Biofilm-Forming Bacteria Count Experiment. CFU counts of MRSA biofilms in the prevention assay after 1 and 3 days of exposure to MRX-4 + gentamicin (C + G) or vancomycin + gentamicin (V + G) loaded on CS or β-TCP/CS beads **(A)**. CFU counts of MRSA biofilms in the killing assay after 1 and 3 days of exposure to MRX-4 + gentamicin (C + G) or vancomycin + gentamicin (V + G) loaded on CS or β-TCP/CS beads **(B)**. *p < 0.05, **p < 0.01, ***p < 0.001, ns, No statistical difference. Data are presented as mean ± SD. One-way ANOVA with Tukey’s test was used.

In the biofilm prevention assay, the control groups (without antibiotic beads, C1; unloaded beads, C2) exhibited high bacterial loads (10^8^–10^9^ CFU/mL). In contrast, the C + G and V + G treatment groups showed a significant reduction in biofilm bacteria by more than 8 log units on both day 1 and day 3 (P < 0.001). No statistically significant differences were observed between CS and β-TCP/CS beads, regardless of the antibiotic combination used, indicating that both carriers were equally effective in biofilm prevention (Figure 2A).

For the biofilm eradication assay, control groups (C1 and C2) again displayed high bacterial loads (approximately 10^8^ CFU/mL) in pre-formed biofilms. After 1 day of exposure to antibiotic-loaded beads (C + G or V + G), bacterial counts within biofilms were reduced by approximately 3–4 log units (P < 0.01). Similar results were observed on day 3, confirming the continued efficacy of both antibiotic combinations. Notably, at day 3, C + G exhibited a significantly greater reduction in biofilm bacteria compared to V + G (P < 0.01), suggesting a superior bactericidal effect. Additionally, no significant differences were observed between CS and β-TCP/CS beads, further reinforcing that the carrier material did not influence antimicrobial efficacy (Figure 2B).

### 3.3 CLSM analysis of biofilms

To visualize biofilm structure, bacterial viability, and treatment effects, CLSM was performed. The CLSM images corroborated the CFU assay results, providing direct evidence of biofilm disruption and bacterial viability.

**FIGURE 3:**
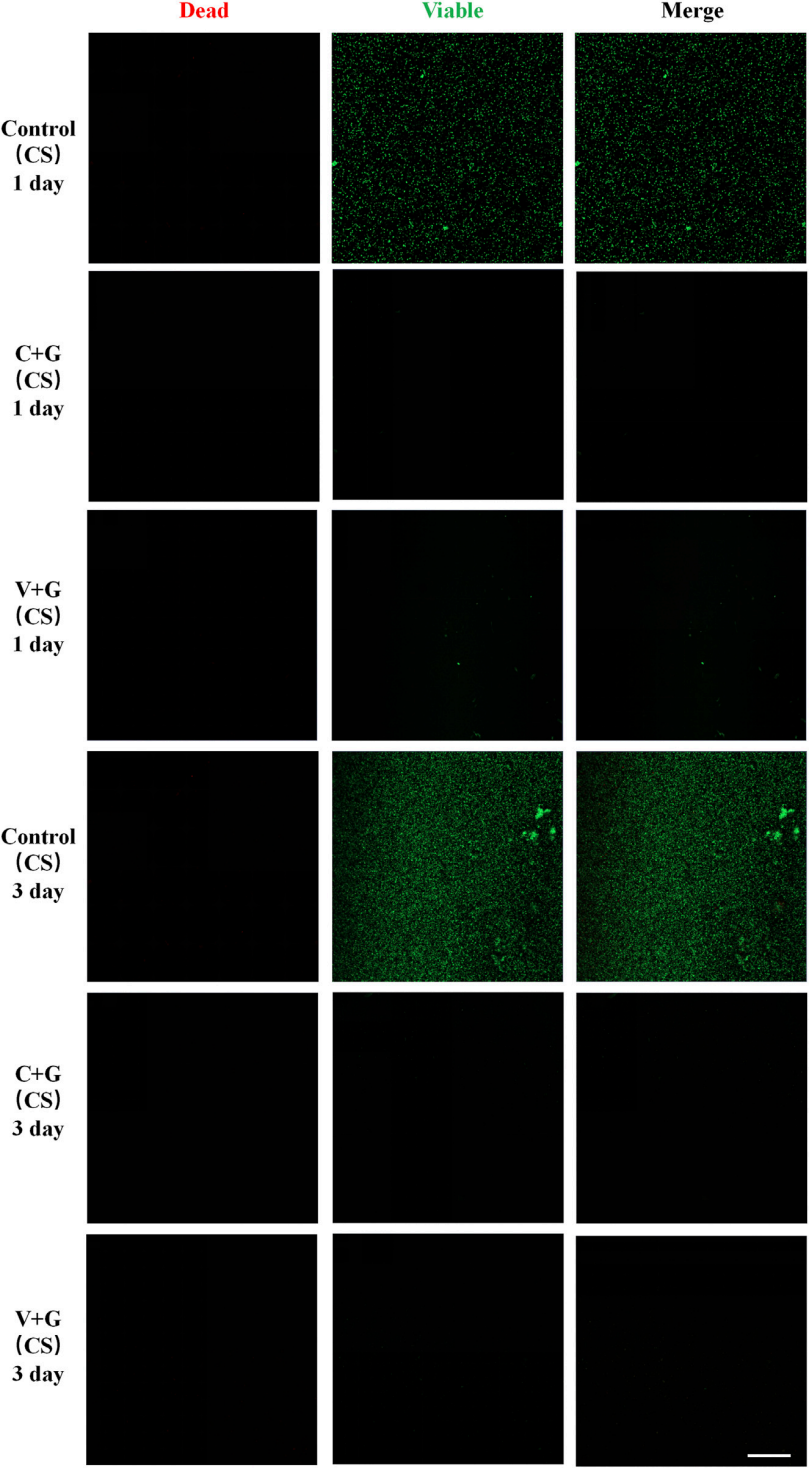
Representative CLSM images of biofilm bacteria in the prevention study (CS), n = 3, scale bar = 50 μm.

**FIGURE 4:**
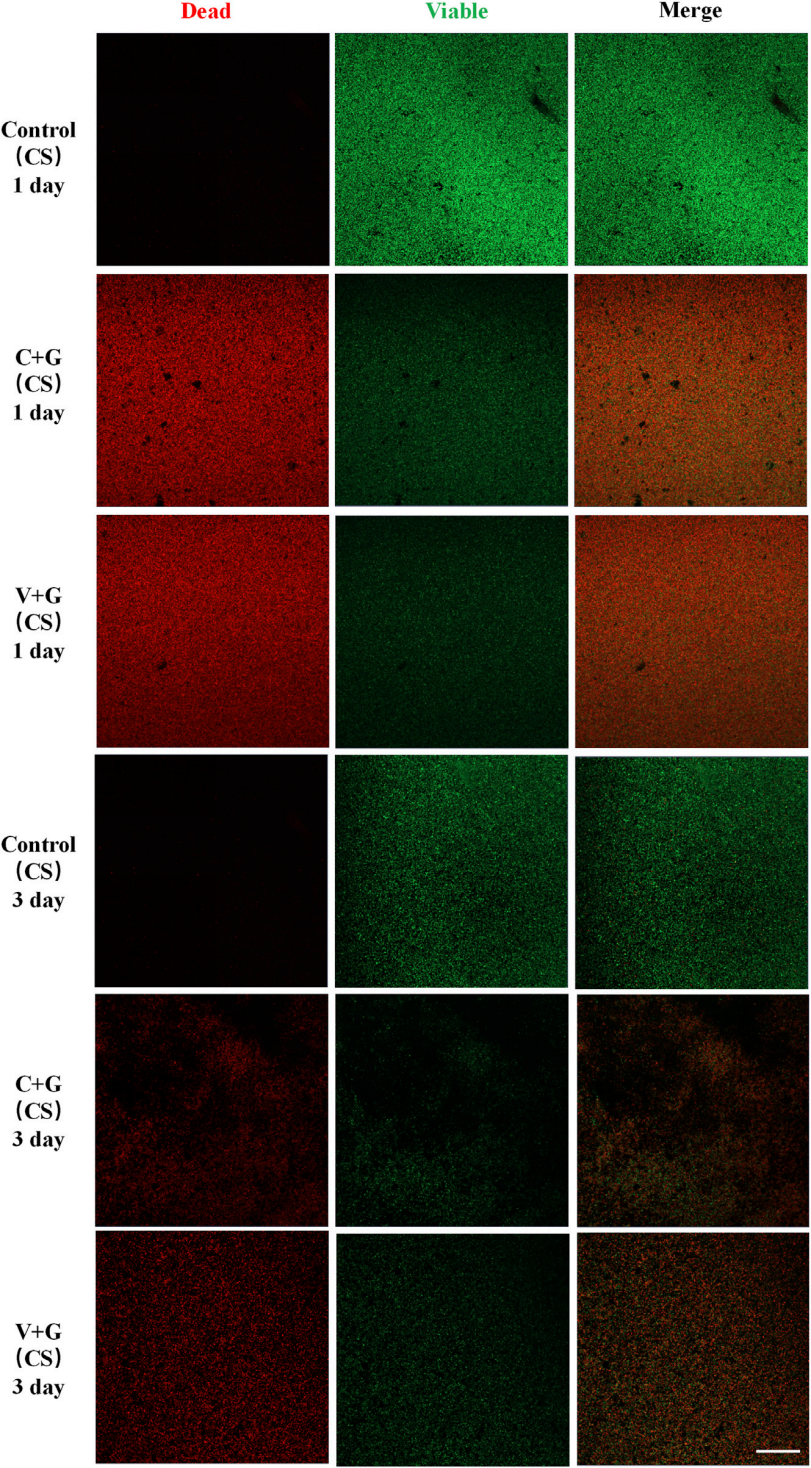
Representative CLSM images of biofilm bacteria in the killing Study (CS), n = 3, scale bar = 50 μm.

In the biofilm prevention assay, the control group exhibited large patches of green fluorescence, indicating extensive biofilm formation with viable bacteria. In contrast, in the antibiotic treatment groups (C + G or V + G, CS beads), only a few scattered green spots were observed, with minimal presence of dead bacteria, suggesting effective biofilm prevention (Figure 3). For the biofilm eradication assay, the control groups again showed large, dense green fluorescence, consistent with viable, established biofilms. However, in the antibiotic-treated groups (C + G or V + G, CS beads), a mixture of green (viable bacteria) and red fluorescence (dead bacteria) was observed, indicating partial bacterial killing within biofilms. The green fluorescence was notably reduced compared to the control group, confirming effective biofilm reduction. Additionally, dead bacteria appeared adhered to the substrate, which was not observed in untreated biofilms, suggesting that bacterial eradication was occurring within the biofilm matrix (Figure 4). Additionally, similar results were observed in β-TCP/CS beads, further confirming that the choice of carrier did not significantly impact antimicrobial efficacy (Supplementary Figures S3, S4).

## 4 Discussion

The treatment of bone infections requires a comprehensive approach that includes surgical debridement, antibiotic therapy, and tissue defect reconstruction (Moriarty et al., 2022). Of these, the appropriate selection and administration of antibiotics are essential for eliminating infections and preventing their recurrence. According to the 2020 International Consensus on Fracture-Related Infection (FRI), the current gold standard for treating FRI involves early empirical antibiotic therapy combined with LADS, followed by pathogen-directed therapy based on microbiological culture results (Depypere et al., 2020). Nonetheless, despite the widespread use of vancomycin and linezolid for treating MRSA-related bone infections, their effectiveness has been increasingly questioned due to rising antimicrobial resistance and associated adverse effects (Cairns et al., 2023; Bender et al., 2018). Therefore, identifying new antibiotics with enhanced safety and efficacy profiles is an urgent priority in clinical research.

In the present study, an MRSA biofilm model was utilized to assess the local antimicrobial efficacy of the novel oxazolidinone derivative MRX-4. β-TCP/CS and CS were employed as local antibiotic carriers, and MRX-4+gentamicin was compared with the traditional vancomycin + gentamicin regimen. The results indicated that MRX-4+gentamicin exhibited significant biofilm-preventive effects, markedly reducing bacterial counts. Additionally, MRX-4 demonstrated superior biofilm penetration and sustained antimicrobial efficacy, surpassing vancomycin in the eradication of pre-formed biofilms, suggesting that MRX-4 may possess enhanced diffusion through EPS or greater intracellular activity against biofilm-embedded bacteria (Flemming et al., 2025). Considering that biofilm formation is a critical factor contributing to the persistence and recurrence of bone infections, this indicates that MRX-4 may offer a therapeutic advantage over traditional antibiotics in localized therapy for chronic osteomyelitis and DFO.

The biofilm formation assays corroborated that the combinations of MRX-4 with gentamicin and vancomycin with gentamicin markedly inhibited the development of MRSA biofilms. Bacterial CFU counts experienced a reduction of approximately 8 log orders in comparison to the control group, thereby affirming the effectiveness of both antibiotic combinations in impeding biofilm formation. Nonetheless, MRX-4 sustained a consistent inhibitory effect throughout the experimental duration, indicating its enduring biofilm-preventive attributes, whereas the efficacy of vancomycin diminished more swiftly. Furthermore, in the biofilm eradication assays, the MRX-4 and gentamicin combination demonstrated significantly superior bacterial reduction at the 3-day mark in contrast to the vancomycin and gentamicin combination, thereby fortifying the hypothesis that MRX-4 might possess greater efficacy in eliminating established MRSA biofilms.

The enduring antimicrobial efficacy of MRX-4 in combination with gentamicin was substantiated through ZOI assays, which demonstrated that MRX-4, when paired with gentamicin, sustained its antimicrobial efficacy for a minimum of 40 days. In contrast, the combination of vancomycin and gentamicin exhibited a marked diminishment in activity after 15 days, and by day 40, its efficacy was nearly negligible (P < 0.05). These findings imply that MRX-4, upon conversion to its active metabolite MRX-1, facilitates a sustained release profile concurrent with the degradation of biomaterials, thereby ensuring a prolonged elevation of local antibiotic concentration. Considering the necessity for prolonged antibiotic treatment in cases of chronic osteomyelitis, the sustained local activity provided by MRX-4 may confer a clinical benefit over traditional vancomycin therapy.

Mechanistically, the observed superiority of MRX-4/MRX-1 over vancomycin in disrupting and penetrating MRSA biofilms may stem from fundamental differences in their diffusion and uptake characteristics. Vancomycin—a large glycopeptide—has well-documented limitations in diffusing through the dense EPS matrix of biofilms, often requiring concentrations up to thousands of times the planktonic MIC to achieve modest eradication (Darouiche et al., 1994). In contrast, MRX-1 (contezolid) is a smaller, more lipophilic oxazolidinone, enabling comparatively easier diffusion into biofilm interiors. Additionally, oxazolidinones like MRX-1 are known to penetrate bacterial cells more efficiently than vancomycin, facilitating inhibition of intracellular protein synthesis—an advantage in targeting biofilm-embedded, slow-growing cells (persisters) (Rowe et al., 2021; Zelmer et al., 2022). Together, these pharmacodynamic distinctions—enhanced diffusion through biofilm EPS and superior intracellular activity—provide a stronger mechanistic rationale for MRX-4’s enhanced biofilm penetration and efficacy compared with vancomycin. Building on this comparison, it is also important to contextualize MRX-4 against other antibiotics currently explored in local delivery systems for osteomyelitis. Linezolid, another oxazolidinone, has demonstrated efficacy against MRSA biofilms, but its long-term systemic use is restricted by hematologic and mitochondrial toxicities (Bongers et al., 2019; Cobb et al., 2020). MRX-4 was specifically designed to maintain oxazolidinone antibacterial activity while improving safety, thereby permitting extended administration. Daptomycin, in contrast, is valued for its rapid bactericidal activity and has been investigated in bone-targeted therapies; however, its effectiveness is diminished in acidic or ion-rich environments such as infected bone tissue, and resistance can emerge during prolonged therapy (Lamp et al., 1992; Fuchs et al., 2001; Marty et al., 2006). By comparison, MRX-4 combines the safety advantages over linezolid with a sustained release profile and reliable biofilm penetration, highlighting its promise as a next-generation agent for localized antibiotic delivery in MRSA osteomyelitis.

To evaluate the impact of antibiotic carrier selection on antimicrobial efficacy, β-TCP/CS and pure CS were employed. The findings indicate that the type of carrier did not significantly affect antibacterial effectiveness. Both β-TCP/CS and CS enabled sustained antibiotic release and yielded comparable outcomes in terms of biofilm inhibition and eradication, consistent with previous studies that have demonstrated the efficacy of CS-based carriers for localized antibiotic delivery (Zhang et al., 2020).

Moreover, β-TCP may offer additional support for bone regeneration, making it a favored option in the management of bone defects (Bohner et al., 2020; Lou et al., 2015). Nevertheless, its elevated cost presents a constraint to its widespread clinical application. Additionally, a comparison was made between MRX-4+gentamicin and monotherapy, and the data indicate that the combination therapy exhibited superior antibacterial efficacy. This effect is likely due to gentamicin’s capacity to inhibit bacterial protein synthesis, thereby enhancing MRX-4-mediated bacterial elimination (Chareyre et al., 2019). Furthermore, gentamicin’s high-water solubility facilitates improved antibiotic diffusion within the carrier, optimizing MRX-1’s local bioavailability (Croitoru et al., 2015). Based on these results, gentamicin was chosen as a synergistic agent to augment MRX-4’s efficacy in biofilm-targeted therapy.

Although this investigation underscores the prospective efficacy of MRX-4 as a localized treatment for MRSA-associated bone infections, it is imperative to recognize several constraints. Initially, the research was confined to a 3-day observation window, with no subsequent evaluations conducted over longer periods, such as 7, 14, or 30 days. Despite the ZOI assays demonstrating sustained antimicrobial efficacy for up to 40 days, further research is warranted to ascertain whether MRX-4 continues to exert its biofilm-disrupting effects over more extended durations. Future research endeavors should encompass more prolonged *in vitro* and *in vivo* experiments to evaluate the long-term efficacy of MRX-4 in chronic infections. Moreover, this study was exclusively conducted *in vitro* utilizing an MRSA biofilm model; however, *in vivo* bone infections encompass additional variables, including host immune responses, vascular supply, and local pH fluctuations, which could impact antibiotic efficacy (Chen et al., 2023). In particular, osteomyelitis represents a highly complex pathological condition involving dynamic host–pathogen interactions, bone remodeling processes, and systemic pharmacokinetics of antibiotics, none of which are adequately reproduced in our *in vitro* biofilm model. These factors can profoundly influence drug penetration, local efficacy, and treatment outcomes in clinical settings, and thus highlight the necessity for carefully designed *in vivo* studies to capture the multifaceted nature of bone infection (W et al., 2021; Billings and Anderson, 2022). In addition, potential toxicity or biocompatibility issues of MRX-4 in bone tissue were not addressed in the present study. Given that local delivery systems must balance antimicrobial potency with host tissue compatibility, it will be essential for future research to investigate MRX-4’s cytocompatibility with osteoblasts, osteoclasts, and surrounding bone matrix to ensure its safety for clinical translation. Therefore, to more comprehensively understand the therapeutic potential of MRX-4, it is advisable to establish animal models of chronic osteomyelitis and diabetic foot infections to verify its pharmacokinetics and clinical applicability. Lastly, while MRX-4 was assessed in conjunction with gentamicin, the study did not explore alternative synergistic antibiotic combinations, such as those involving linezolid or daptomycin. In light of the intricate nature of MRSA biofilms, future investigations should examine additional antibiotic combinations to refine local treatment protocols and mitigate the emergence of resistance.

Overall, this study demonstrates the novel and significant finding that MRX-4 combined with gentamicin exhibits high efficacy in both preventing and eradicating MRSA biofilms, highlighting its potential as a new local therapeutic agent for osteomyelitis and diabetic foot bone infections. Importantly, MRX-4 showed a sustained release profile and superior biofilm penetration, which clearly differentiates it from vancomycin and underscores its promise as a next-generation alternative in localized antibiotic delivery systems. Furthermore, our results provide the first evidence that both β-TCP/CS and CS are effective carriers for MRX-4 delivery, thereby expanding their potential for clinical application beyond traditional agents. These findings represent a meaningful step forward in developing more effective localized strategies against biofilm-related bone infections. Nevertheless, given the short experimental timeframe and lack of *in vivo* validation, further long-term studies and animal experiments will be essential to fully establish MRX-4’s clinical efficacy and safety. Future research should therefore focus on extended treatment periods, alternative antibiotic combinations, and *in vivo* pharmacokinetic assessments to refine MRX-4-based localized therapies for chronic bone infections.

## 5 Conclusion

This study demonstrated that MRX-4 combined with gentamicin, delivered via biodegradable CS and β-TCP/CS carriers, effectively prevented and eradicated MRSA biofilms while maintaining sustained antimicrobial activity for at least 40 days. Compared to vancomycin + gentamicin, MRX-4 exhibited superior biofilm penetration and prolonged antibacterial effects, making it a promising alternative for localized osteomyelitis and DFO treatment. Both CS and β-TCP/CS proved to be effective antibiotic carriers, supporting stable drug release. These findings highlight the potential of MRX-4 for local antibiotic delivery, warranting further research to validate its long-term efficacy and clinical application.

## Data Availability

The raw data supporting the conclusions of this article will be made available by the authors, without undue reservation.

## Supplementary material

SUPPLEMENTARY FIGURE S1 ZOIs of CS versus β-TCP/CS by C + G and V + G. Statistical diagram comparing ZOI sizes between CS and β-TCP/CS over time **(A,B)**.

SUPPLEMENTARY FIGURE S2 Representative CLSM images of biofilm bacteria in the prevention study (β-TCP/CS), n = 3, scale bar = 50 μm.

SUPPLEMENTARY FIGURE S3 Representative CLSM images of biofilm bacteria in the killing Study (β-TCP/CS), n = 3, scale bar = 50 μm.
