# Supplementary figures and images for: β-tricalcium phosphate/calcium sulfate loaded with contezolid acefosamil (MRX-4) for antimicrobial potency, prevention and killing efficacy of MRSA biofilm
Source: Front Pharmacol. 2025 Sep 23;16:1657099. doi: 10.3389/fphar.2025.1657099 (PMC12501469; doi:10.3389/fphar.2025.1657099)

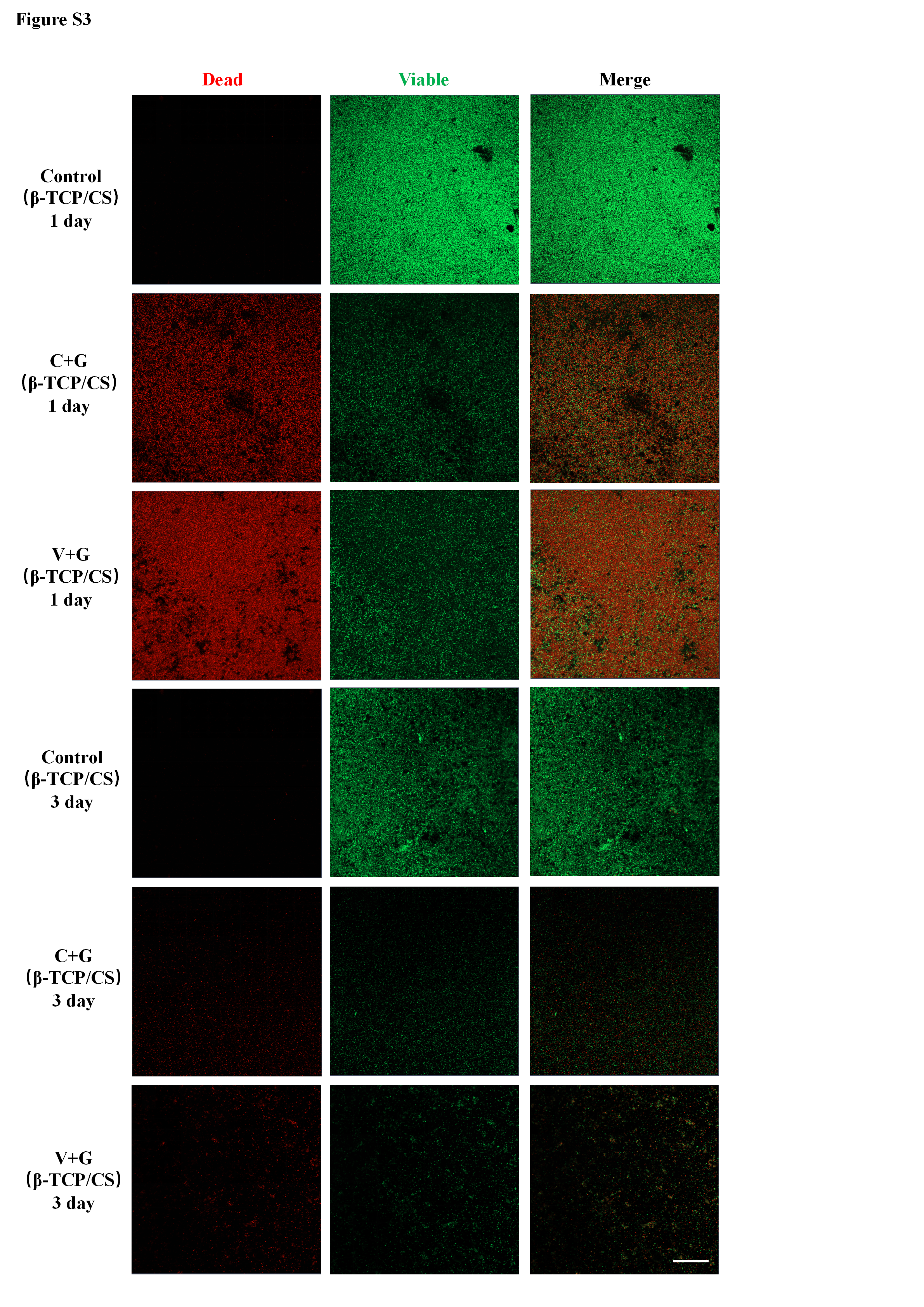

Supplement: Supplementary file 1 [file Image3.tif]

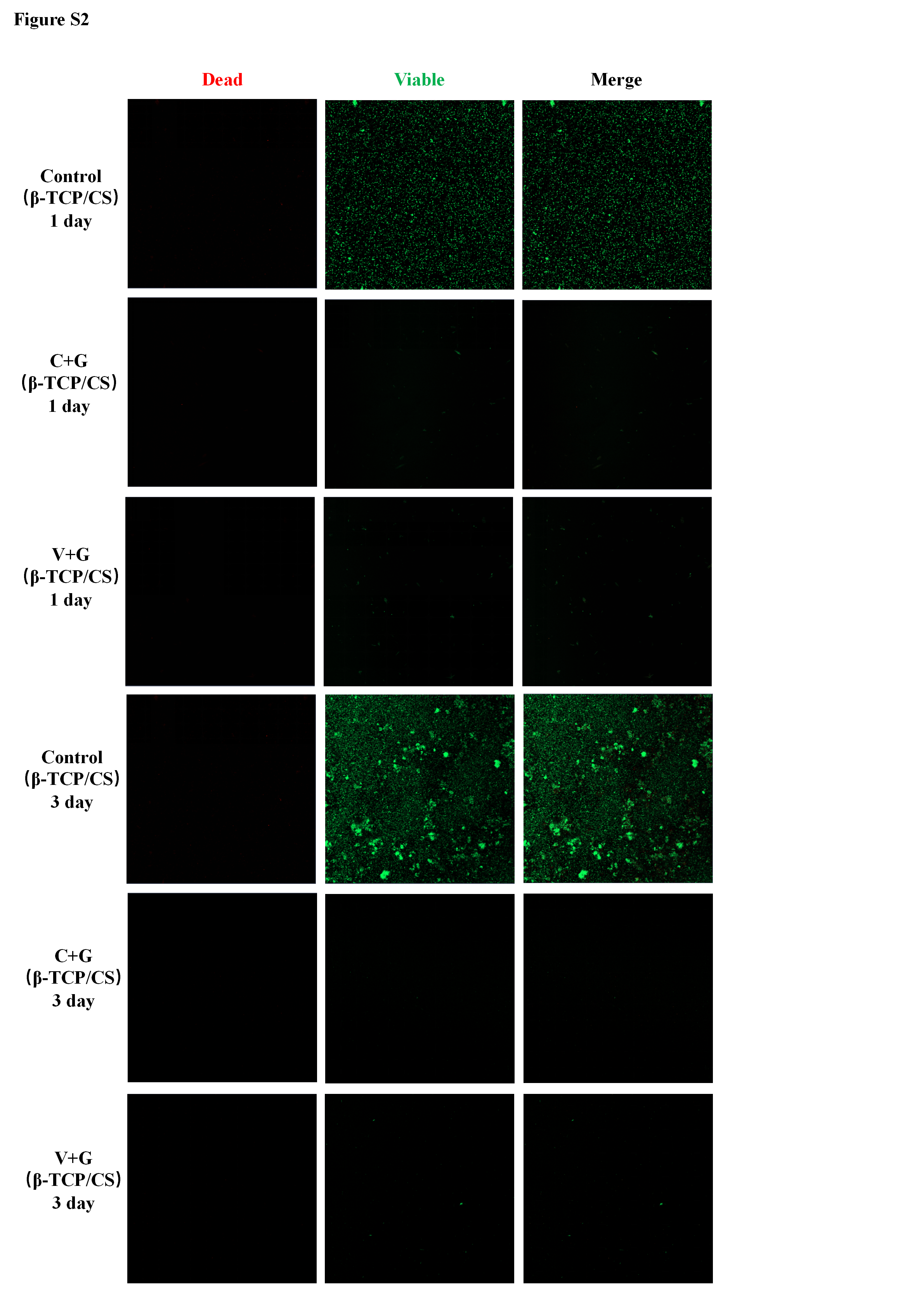

Supplement: Supplementary file 2 [file Image2.tif]

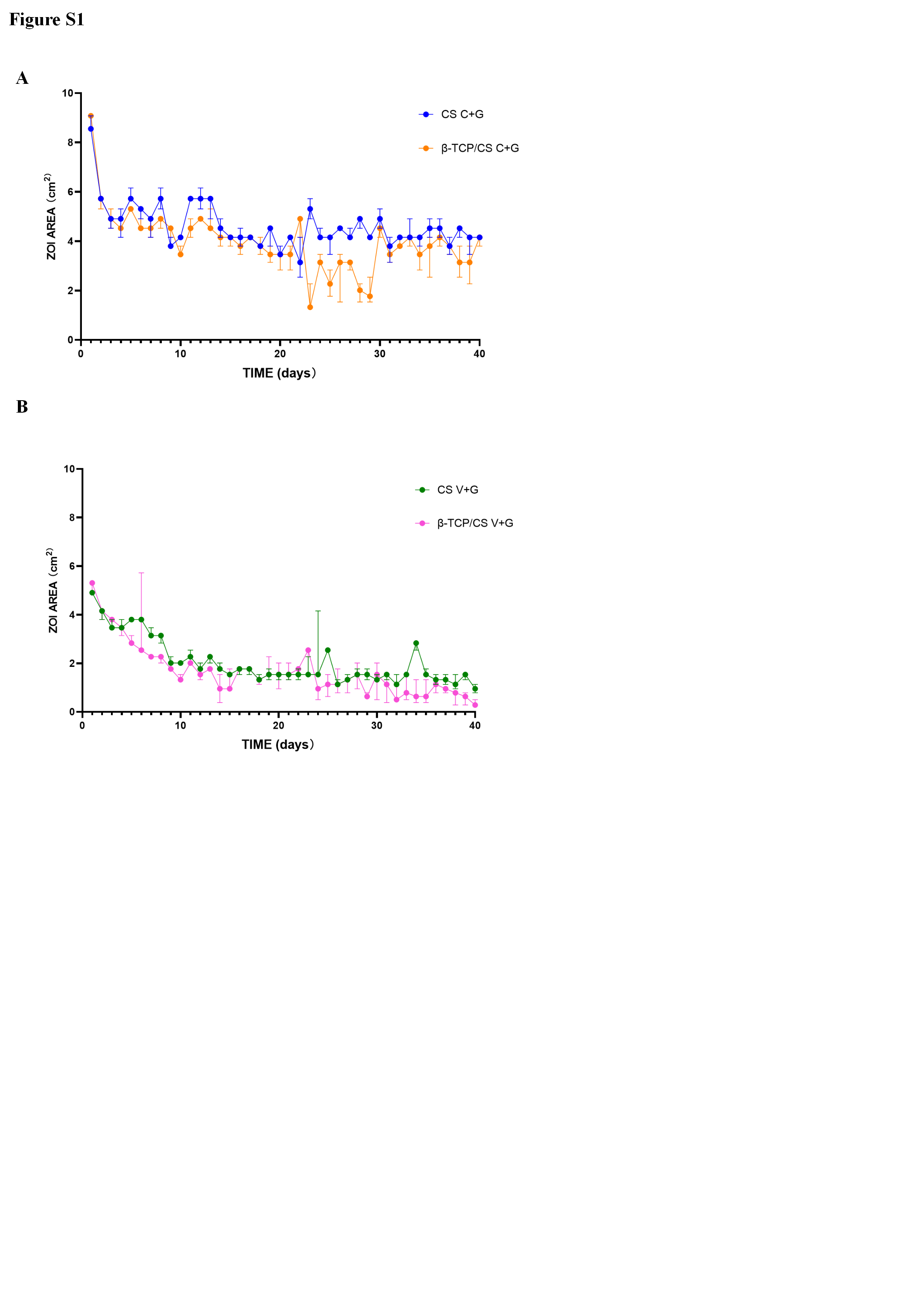

Supplement: Supplementary file 3 [file Image1.tif]
